# Evaluation of early ocular discomfort after glaucoma surgery: trabeculectomy versus Ahmed glaucoma valve implantation

**DOI:** 10.5935/0004-2749.2023-0033

**Published:** 2024-03-05

**Authors:** Ricardo Y. Abe, Douglas R. Yamamoto, Fernanda Maria Silveira Souto, José Paulo Cabral Vasconcellos, Vital Paulino Costa

**Affiliations:** 1 Department of Ophthalmology, Universidade Estadual de Campinas, Campinas, SP, Brazil; 2 Hospital Oftalmológico de Brasília, Brasília, DF, Brazil

**Keywords:** Glaucoma/surgery, Paresthesia, Trabeculectomy, Glaucoma drainage implants, Postoperative care

## Abstract

**Purpose:**

This study aims to compare the initial ocular discomfort symptoms resulting from trabeculectomy and Ahmed glaucoma valve implantation surgeries.

**Methods:**

A prospective comparative study was conducted. The evaluation of ocular discomfort employed a questionnaire designed to identify the frequency and severity of distinct symptoms: ocular pain, general discomfort, tearing, foreign body sensation, and burning. This questionnaire was administered prior to surgery as a baseline, and subsequently at 7, 30, and 90 days post-surgery. Simultaneously, the Ocular Surface Disease Index (OSDI) was applied at these same time intervals.

**Results:**

The study encompassed a total of 17 patients (9 undergoing trabeculectomy and 8 undergoing Ahmed glaucoma valve implantation). The Ahmed glaucoma valve implantation group exhibited higher tearing levels at baseline (p=0.038). However, no statistically significant differences in symptoms were observed between the two surgeries at 7 and 30 days post-surgery. At the 90-day mark following surgery, patients who had undergone trabeculectomy reported a significantly higher foreign body sensation (p=0.004). Although OSDI scores did not differ between groups at baseline, the trabeculectomy group showed significantly higher OSDI scores than the Ahmed glaucoma valve implantation group at 7, 30, and 90 days after surgery (p<0.05).

**Conclusion:**

Post-surgery, patients who had undergone trabeculectomy experienced increased foreign body sensation. Trabeculectomy appears to cause greater early postoperative ocular discomfort compared to the Ahmed glaucoma valve implantation group.

## INTRODUCTION

Glaucoma stands as the leading cause of irreversible blindness across the globe and is projected to impact approximately 111.8 million individuals by the year 2040^(1)^. While the majority of patients can be treated through clinical therapy and laser procedures, certain cases require surgical interventions to mitigate intraocular pressures (IOP) and prevent deterioration of the visual field.

The most commonly performed surgical procedure for glaucoma is trabeculectomy^(2)^. This technique holds the benefit of achieving low postoperative IOP levels and distinguishes itself through its cost-effectiveness compared to alternative procedures^(3)^. In contrast, the implantation of glaucoma drainage devices has been increasing yearly^(2)^. Among these devices, the Ahmed glaucoma valve offers the advantage of reducing the occurrence of hypotony due to its valve-restrictive mechanism.

During glaucoma surgery, the primary objective is to attain the target IOP, aiming to avoid disease progression while upholding the patient’s quality of life^(4)^. Unfortunately, even upon reaching the target IOP, patients may experience postoperative ocular discomfort, expressing concerns about sensations of burning, foreign body sensation, pain, and tearing – a cluster of ocular symptoms commonly referred to as filtering bleb dysesthesia^(5)^.

Despite the initial description of bleb dysesthesia following trabeculectomy, no investigations have been conducted among patients who undergo aqueous shunt implantation. Therefore, within this prospective study, we compared the occurrence of early ocular discomfort symptoms between patients who received primary trabeculectomy and those who underwent primary Ahmed glaucoma valve implantation.

## METHODS

This study adopted a prospective comparative case series design. Participants were recruited from the Department of Ophthalmology at the *Hospital das Clínicas* – University of Campinas – UNICAMP. The study protocol underwent thorough review and approval by the Institutional Review Board. All patients signed an informed consent and the study complied with the Declaration of Helsinki guidelines for human subject research. During follow-up, subjects underwent comprehensive ophthalmologic examinations including review of medical history, visual acuity, slit-lamp biomicroscopy, IOP measurement (Goldmann tonometer), gonioscopy (Gonio Lens Posner), dilated fundoscopic examination with 78 diopters lens, and optic disc photography.

To evaluate ocular discomfort, we utilized a questionnaire designed to identify both the frequency and severity of different symptoms: ocular pain, general discomfort, tearing, foreign body sensation, and burning, similar to previous study^(5)^. For frequency, we used a linear scale ranging from 0 to 4, where 0 denoted no occurrence, 1 indicated sporadic occurrence, 2 represented occurrence half the time, 3 signified occurrence most of the time, and 4 marked constant occurrence. As for severity, a linear scale ranging from 1 to 4 was used, where 1 indicated mild symptoms, 2 moderate symptoms, 3 severe symptoms, and 4 very severe symptoms. This questionnaire was administered prior to surgery at baseline, as well as at the intervals of 7, 30, and 90 days post-surgery. To mitigate potential bias arising from ocular surface disease, a frequent issue among glaucoma patients due to the effects of glaucoma medications, the Ocular Surface Disease Index (OSDI) was also applied at these same time points^(6)^.

Skilled glaucoma specialists (DRY and FMS) conducted all surgical procedures in accordance with standardized surgical protocols^(7,8)^. The surgeries were performed under peribulbar anesthesia using 2% lidocaine. In the case of trabeculectomy, a 6-0 silk traction suture was placed in the cornea adjacent to the limbus superiorly. Preparation involved creating a fornix-based conjunctival flap centered at 12 o’clock, followed by dissection of the conjunctiva and Tenon’s capsule posteriorly. A sponge soaked in 0.1-0.2 ml of mitomycin C (0.2 mg/ml) was then applied under the conjunctiva for 3 minutes, and the area was subsequently rinsed meticulously with balanced salt solution (20 ml). A square scleral flap appro ximately half in thickness (measuring about 4 x 4 mm) was dissected just into clear cornea. Before entering the anterior chamber, a paracentesis was performed temporally. The procedure included resection of an anterior corneo-trabecular block (approximately 1.5 mm) and a peripheral iridectomy. The scleral flap was sutured using two 10-0 nylon sutures positioned at the flap’s corners. Balanced salt solution was introduced into the anterior chamber via the paracentesis, and the aqueous flow under the scleral flap was observed. If the flow was deemed excessive, additional sutures were applied to the scleral flap. Closure of the conjunctiva and Tenon’s capsule involved the use of three nylon 10-0 sutures anchored at the limbus. At the end of the procedure, the conjunctival closure was examined for water-tightness. Regarding the Ahmed glaucoma valve implantation, a fornix-based conjunctival flap was created preferably in the superotemporal quadrant. Priming of the Ahmed glaucoma valve was accomplished by flushing balanced salt solution through the tube to confirm its patency. The anterior edge of the plate was anchored to the sclera using 9-0 nylon sutures, ensuring a distance of at least 8 mm from the limbus. To access the anterior chamber, a 23-gauge needle was inserted 1 mm posterior to the limbus to establish a track. A rectangular donor scleral patch graft (4 × 6 mm) was fashioned and sutured over the tube using 10-0 nylon sutures. The conjunctiva was secured using similar 10-0 nylon sutures.

## RESULTS

A total of 17 patients (9 in the trabeculectomy group and 8 in the Ahmed glaucoma valve implantation group) were enrolled. The clinical and demographic characteristics for both groups are detailed in table 1. Patients in the Ahmed glaucoma valve implantation group exhibited significantly younger ages (p=0.008) and a higher frequency of neovascular glaucoma (p=0.012). Comparisons between baseline and postoperative measurements revealed significant reductions in both IOP and the number of prescribed eye drops (Table 2). No surgical complications were observed during follow-up. We multiplied the values of frequency and severity to obtain a final score for each symptom (Table 3). To compare the symptoms in each group, the Wilcoxon rank-sum test was used, chosen due to the non-parametric distribution of these variables.

**Table 1:** Clinical and demographic variables of patients included in the study

Parameters	Trabeculectomy	Ahmed glaucoma valve	p-value
Age, years	64.6 ± 4.4	46.1 ± 5.3	0.008
Gender, % female	22.2	37.5	0.153
Race			
% White	75	77.7	1.000
% Black	25	22.2	
Etiology			
Primary open angle glaucoma	62.5%	11.1%	0.012
Primary angle closure glaucoma	25%	0	
Neovascular glaucoma	0	55.5%	
Steroid-induced glaucoma	0	11.1%	
Uveitic glaucoma	12.5%	22.2%	
Number of glaucoma eye drops	3.75 ± 0.16	3.33 ± 0.23	0.177

**Table 2:** Comparison between baseline and postoperative visual acuity, intraocular pressure, and glaucoma medications

	Trabeculectomy			Ahmed glaucoma valve implantation		
Parameters	Baseline	Postoperative	p-value	Baseline	Postoperative	p-value
Visual acuity (logMar)						
Mean ± SD	0.46 ± 0.17	0.56 **±** 0.24	0.575	1.16 ± 0.54	1.21 ± 0.39	0.363
IOP (mmHg)						
Mean ± SD	26.2 ± 3.41	12.6 **±** 1.07	0.027	35.33 ± 3.67	13.33 ± 2.24	0.003
Glaucoma eye drops						
Mean ± SD	3.8 ± 0.2	0.4 ± 0.24	<0.001	3.5 ± 0.22	1.83 ± 0.65	0.067

SD= standard deviation; IOP= intraocular pressure.

**Table 3:** Comparison of ocular symptoms before and after surgery

Time	Variables	Trabeculectomy (9 patients)	Ahmed glaucoma valve (8 patients)	p-value
**Baseline**	Ocular discomfort	0.1	2.8	0.120
	Ocular pain	1.75	5	0.676
	Tearing	0	2	**0.038**
	Burning	0	2.3	0.082
	Foreign body sensation	1.5	0.44	0.280
	OSDI score	33	28.8	0.561
**7 days after surgery**	Ocular discomfort	5.5	5.6	0.527
	Ocular pain	3.6	5.6	0.961
	Tearing	3	3.5	0.767
	Burning	0.25	1.5	0.306
	Foreign body sensation	5.6	4.2	0.357
	OSDI score	52.4	39.0	**0.004**
**30 days after surgery**	Ocular discomfort	3.7	2.5	0.170
	Ocular pain	2.7	3.5	0.556
	Tearing	1.75	2	0.566
	Burning	0	1	0.169
	Foreign body sensation	3.6	2.4	0.155
	OSDI score	49	35.1	**0.009**
**90 days after surgery**	Ocular discomfort	2.75	1.6	0.124
	Ocular pain	2.5	2.1	0.279
	Tearing	1.3	1	0.778
	Burning	0	0.7	0.169
	Foreign body sensation	3.2	0.7	**0.004**
	OSDI score	40.2	20.6	**0.026**

We observed an increased incidence of tearing in the Ahmed glaucoma valve group at the baseline (p=0.038) (Table 3). However, at both the 7-day and 30-day post-surgery marks, no significant difference in symptoms was evident between the surgeries. Notably, 90 days after the surgery, patients who had undergone trabeculectomy showed significantly increased foreign body sensation (p = 0.004). Although no difference in OSD1 scores was detected between the groups at baseline, the trabeculectomy group exhibited significantly higher OSD1 scores than the Ahmed glaucoma valve group at 7, 30, and 90 days after the surgery (p<0.05).

## DISCUSSION

In this prospective comparative case series study, it was observed that patients who underwent trabeculectomy encountered greater early ocular discomfort post-surgery than those who received Ahmed glaucoma valve implantation. A distinct observation was that patients subjected to trabeculectomy reported increased foreign body sensation 90 days after the surgery. Furthermore, the trabeculectomy group presented significantly worse OSD1 scores 7, 30, and 90 days post-surgery when compared to the Ahmed glaucoma valve implantation group.

Budenz et al. introduced the term “bleb dysesthesia” to characterize ocular discomfort following trabeculectomy^(5)^. They established that ocular discomfort (manifested as ocular pain, tearing, foreign body sensation, and burning) can be prevalent, affecting approximately 67% of eyes (with a mean time of 28.3 months between trabeculectomy and questionnaire administration) after the surgery. Their findings additionally noted that discomfort tends to arise more frequently from blebs not covered by the superior eyelid and those situated in the superior-nasal region. Recognizing these symptoms during the postoperative period following glaucoma incisional surgery holds significance in ensuring patient’s everyday well-being and quality of life. Although trabe-culectomy remains the predominant glaucoma incisional procedure, the use of aqueous shunts is increasing^(2)^. However, no previous studies had undertaken a comparison of postoperative dysesthesia between trabeculectomy and aqueous shunt procedures.

Aqueous shunt devices, including the Ahmed glaucoma valve, rely on the aqueous flow through the tube, plate, and subconjunctival space. The formation of a bleb around the plate, as well as the plate itself or the scleral patch graft that covers the tube’s entry into the anterior chamber, could potentially lead to postoperative discomfort for patients, akin to what is observed in patients undergoing trabeculectomy. However, our observations revealed that patients who underwent trabeculectomy reported a higher incidence of foreign body sensation than the Ahmed glaucoma valve group at the 90-day postoperative stage (p=0.004). Additionally, the trabeculectomy group presented significantly higher OSDI scores compared to the Ahmed glaucoma valve group 7, 30, and 90 days post-surgery (p<0.05).

This phenomenon might find its explanation in the connection between ocular discomfort after glaucoma surgery and corneal sensitivity. In fact, Sanders et al. highlighted that superonasal blebs originating from trabeculectomy demonstrated an increased correlation with corneal dellen^(9)^. Therefore, our hypothesis is that eyes subjected to aqueous shunt devices are inclined to exhibit a reduced incidence of corneal dellen, largely due to the bleb’s posterior positioning, distant from the cornea. Additionally, the placement of the plate in aqueous devices typically ensures coverage by the eyelid, thereby minimizing the risk of ocular discomfort.

This study presents certain limitations. First, as the purpose was to include patients undergoing primary surgical procedures, baseline statistical differences were evident in terms of glaucoma etiology and age between groups, given that patients undergoing Ahmed glaucoma valve implantation pertain to the category of secondary glaucoma, often associated with a higher likelihood of trabeculectomy failure. Despite the direct connection between the use of eye drops and ocular surface discomfort, it is noteworthy that 90 days post-surgery, patients who received Ahmed glaucoma valve implantation were using more eye drops (1.83 ± 0.65) compared to the trabeculectomy group (0.4 ± 0.24). However, patients subjected to trabeculectomy exhibited increased foreign body sensation and worse OSDI scores, suggesting the potential dominance of bleb dysesthesia over ocular surface symptoms stemming from eye drop use. Second, the small sample could impede the generalization of outcomes; yet, the investigation’s significance exploring bleb-related dysesthesia in the context of trabeculectomy and Ahmed glaucoma valve implantation is underscored. Third, the study did not incorporate visual field data due to the presence of low visual acuity at baseline in numerous Ahmed glaucoma valve group patients, rendering their visual field results unreliable.

Despite the findings of this case series report, we believe that further studies are imperative to delve into ocular dysesthesia across different types of glaucoma surgery, it is important to comprehend the circumstances under which given the increased prevalence of ocular surface disease in the elderly and the potential for glaucoma eye drop usage to induce ocular discomfort, comprehending the circumstances under which a glaucoma surgical procedure might aggravate the ocular discomfort^(10)^.
